# Gold as a Filling Material

**Published:** 1905-12-15

**Authors:** J. L. Sweetnam


### GOLD AS A FILLING MATERIAL.

BY J. L. SWEETNAM, D.D.S.

Read before the Michigan State Dental Association, July 12th, 1905.

This subject is assigned to me for a short paper for your consideration to-day. Having stowed away twenty pounds of gold during the past twenty-five years I am glad to have this subject. Gold is the most valuable material that has ever been used for the purpose of filling teeth, I say the most valuable advisedly, for no other material has the wide range of usefulness possessed by gold. Unfortunately it is also the most abused filling material we possess.

When we stop to consider the high order of manipulative skill required to produce good results with gold, how little wonder we are constantly striving to find something to take its place. This effort has not been entirely wasted for because of it other materials have been found with limited ranges of usefulness.

I do not consider it nesessary for me at this time to say anything about the physical properties of gold, so will confine my remarks to some conditions which need to be carefully discussed.

First, more time should be devoted to the training of the hands to properly manipulate gold so as to produce fillings that will resist the great strains to which they are subjected.

Second: The underlying principles of the formation of cavities to be filled with gold need continual study.

Thirdly: The different forms of gold used in filling teeth can be studied by individuals with great profit.

I said in the first place that more time should be devoted to training the hands to properly manipulate gold to a texture that will resist the great strain to which fillings are subjected. Have you ever stood by watching others put gold into a cavity, and felt sorry for them? I have, not once but scores of times, sometimes I have taken their instruments, squared their work and started them over, but to no purpose, they had not the capacity they should have acquired before starting practice. To play well a musical instrument requires early training. If you will permit me to cite an illustration. I have a daughter fourteen years of age, who plays the violin beautifully, having started to practice at seven. If she makes an error I can detect it at once, for I understand music, and play other instruments but I could not take her violin and produce any music. I have had assistants, graduates of our best schools of dentistry, say in dispair, what seems easy to you is impossible with me. I believe this is largely due to a lack of early training, you may now readily see that my argument is; gold has no specific value in itself as a filling material, its usefulness depends upon the skill of the operator to so adapt it to the walls of cavities that it effectually excludes the active influence of decay producing agencies. If this is true, it is equally important that we have the walls of cavities so shaped that we may adapt it more securely. This brings us to the second argument. The underlying principles of the formation of cavities to be filled with gold, are, that it is absolutely necessary to have good strong walls to build against and a flat or secure foundation to build upon, a concave surface will not answer. The base of the filling must be flat, both on account of the filling and on account of the tooth, and every part of the cavity must be accessable to manipulation. If all portions of the filling are to be properly condensed the operator must be able to get at them. We too often try to do the impossible. A properly constructed separator should be placed between the teeth during the packing of gold in proximal cavities whether it is needed for the purpose of separation or not, which it generally is, it is needed to study the tooth being filled and assists giving a clearer view and a freer operating space. Let us briefly consider the different forms of gold used in filling of teeth under the three general heads of cohesive, non-cohesive and crystal or mat gold. Everyone should know something about the merits and demerits of each.

I want to make the broad assertion that no man who has become eminent as a successful conservator of teeth ever built his reputation on crystal gold; and yet there are dentists all over this land trying to fill teeth with this stuff. Some use it in the bottom of cavities, claiming it is easier to start with. There is no easy way to insert good gold fillings, and I condemn every practice of that sort as a makeshift that will end in disastrous failure in time.

Now and then we run across an advocate of non-cohesive gold, who will tell us what wonderful things were done with it, in the good old days before cohesive gold was known. They have extracted teeth that had been filled for "forty-six years" with non-cohesive gold. Generally it will be found that any old thing would have preserved these same teeth just as well. Non-cohesive gold does not have the necessary strength for the average filling; but it may have a useful place, which in my judgment is very limited. If it is used at all, it should be of quality that will become cohesive by annealing when used; in other words have it clean and free from contamination of any sort. Any cohesive gold will become non-cohesive by subjecting it to the fumes of ammonia for ten or fifteen minutes. Then if wanted cohesive again, you have only to drive off the gas with heat and you know that you are using a clean gold. Cohesive gold makes the best fillings when it is properly condensed with finely serated slightly convexed faced pluggers. Such fillings can be inserted as will stand all the strain they will ever be subjected to in the mouth.

Careful attention should be given to the annealing of gold. If the electric annealer cannot be used for lack of current, a piece of mica over a spirit lamp will do just as well. Keep your gold clean and make sure it is thoroughly cohesive. A good deal is said relative to the advantages of hand pressure over mallet pressure. To my mind there is no necessity for conflict between the two methods of condensation, they are both necessary in almost every filling. Personally I prefer the hand mallet in the hands of a well trained assistant, then the amount of hand pressure that is required will not be over-fatiguing to the operator.

The gold should be placed where desired in the cavity and condensed, and we not depend upon any spreading under the plugger by any force that can safely be put upon it. The result after finishing a perfectly packed gold filling should be a surface like a mirror.

The greatest value of gold as applied to children's teeth is not to use it at all. If our mission as dentists is the preservation of the natural teeth, we must not put gold in children's teeth. I would make it an offense punishable by law for a dentist to ever insert gold in the teeth of patients under eighteen years of age. If possible would like to emphasize this with more force. It is cruelty to our little patients and it is worse than useless in their teeth. If the truth, as we know it, were told our patients they would not demand it, for the teeth are not dense enough to stand the strain necessary to properly condense the gold. If it could be properly condensed it would not save the teeth, for the reason that the tubuli of the dentine are so large that moisture will get in around the filling in time and you all know the result.

To sum up then the value of gold as a filling material depends upon three things. Proper formation of cavity, quality of gold, and skill in manipulation. If these matters are properly attended to, I would say that no other material has the value of gold as a filling material.

